# *Nanos* Is Expressed in Somatic and Germline Tissue during Larval and Post-Larval Development of the Annelid *Alitta virens*

**DOI:** 10.3390/genes13020270

**Published:** 2022-01-29

**Authors:** Roman P. Kostyuchenko

**Affiliations:** Department of Embryology, St. Petersburg State University, 199034 St. Petersburg, Russia; r.kostyuchenko@spbu.ru

**Keywords:** annelids, *Nereis*, mesoderm, primordial germ cells, PGCs migration, somatic stem cells, neural system, posterior growth zone, foregut, hindgut, regeneration, evolution

## Abstract

Nanos is a translational regulator that is involved in germline development in a number of diverse animals and is also involved in somatic patterning in several model organisms, including insects. Neither germline development nor somatic stem cell lines/undifferentiated multipotent cells have been characterized in the development of the annelid *Alitta virens*, nor is the mechanism of germ/stem-line specification generally well-understood in annelids. Here, I have cloned an *Avi-nanos* ortholog from *A. virens* and determined the spatial and temporal expression of Nanos. The results revealed that transcripts of *nanos* are expressed during differentiation of multiple tissues, including those that are derived from the 2d and 4d cells. In late embryonic stages and during larval development, these transcripts are expressed in the presumptive brain, ventral nerve cord, mesodermal bands, putative primordial germ cells (PGCs), and developing foregut and hindgut. During metamorphosis of the nectochaete larva into a juvenile worm, a posterior growth zone consisting of *nanos*-positive cells is established, and the PGCs begin to migrate. Later, the PGCs stop migrating and form a cluster of four *nanos*-expressing cells located immediately behind the jaws (segments 4–5). During posterior regeneration following caudal amputation, a robust *Avi-nanos* expression appears de novo at the site of injury and further accompanies all steps of regeneration. The obtained data suggest that blastemal cells are mostly derived from cells of the segment adjacent to the amputation site; this is consistent with the idea that the cluster of PGCs do not participate in regeneration.

## 1. Introduction

*Nanos* was originally discovered and studied in *Drosophila melanogaster* [1]. Its homologs encode proteins belonging to a highly conserved protein family found in both vertebrates and invertebrates. One to four *nanos* genes have been identified across all investigated animal species [2]. Nanos is a translational repressor characterized by a C-terminal domain comprised of two CCHC zinc finger motifs [2].

Expression of this factor is associated with the germline across the Metazoa, and in some cases, Nanos function has been shown to be required for germline development [3,4,5,6,7,8]. However, based on expression patterns, this gene appears to have multiple roles during embryonic and larval development. Indeed, *nanos* genes have been shown to play important roles not only in germline development, but also in somatic development [9,10,11]. Together with *piwi*, *vasa* and some other genes, *nanos* is well known as a member of the germline multipotency program (GMP) genes and a strong molecular marker for the multipotent state of cells [12]. De novo expression of this gene, for example in the regenerative blastema, may indicate local cell dedifferentiation [11,13,14,15,16,17].

Among lophotrochozoans, *nanos*-related genes show variability in their pattern of expression. In the snail *Tritia obsoleta* (*Ilyanassa obsolete*), mRNA of the *nanos* gene becomes restricted to the 4d mesoderm lineage. An experiment using morpholino to knockdown *nanos* resulted in loss of mesodermal and endodermal tissues in the animal [18]. In contrast to *Tritia*, the vetigastropod *Haliotis asinine* expresses a *nanos* homolog in other stages in addition to the early embryonic stage. *HasNanos* expression is detectable during embryonic and larval development, and, being maternally expressed, it is localized to the micromere cell lineages during early cleavage. Later, *HasNanos* becomes progressively more prolific in the dorsal quadrant (D) of the embryo. When the trochophore develops, *HasNanos* mRNA is shown to be expressed in the putative mesodermal bands of the larva and in the primordial germ cells (PGCs) [19]. Among annelids, *nanos* orthologs are expressed in the germline of the leech *Helobdella robusta* [20], during gametogenesis in *Typosyllis antoni* [21], and in PGCs in the polychaetes *Platynereis dumerilii* and *Capitella sp. I* [22,23]. These and other studies also report *nanos* expression in various somatic tissues. For example, in the leech *H. robusta*, *nanos* expression is detectable in ectodermal stem cells, and *Pdu-nanos* and *CapI-nanos* are expressed in multiple somatic tissues, including brain, foregut, mesodermal bands, and the growth zone [20,22,23,24]. Further, de novo expression of *Pdu-nanos* has been reported in regenerating *P. dumerilii* worms [25].

This study examines *nanos*-related genes in *Alitta virens* (formerly *Nereis virens*), a member of the phylum Annelida. Although both early development and regenerative processes in this animal are well-characterized at the morphological level [26,27,28,29,30,31], the initial stages of germline development and behavior of the PGCs during larval and post-larval development (including regeneration) have not been studied. The use of the *nanos* gene as a clear molecular marker can be very useful in describing the development of the PGCs. Furthermore, the study of this gene’s expression during regeneration will provide additional knowledge about possible cell sources during regrowth of lost body parts in *A. virens*. Thus, in order to begin to investigate the embryonic origin(s) of germline and somatic stem cell lines/undifferentiated multipotent cells that have not been characterized in the annelid *A. virens*, I have cloned a *nanos* ortholog (*Avi-nanos*) and examined its developmental expression patterns by whole-mount in situ hybridization during embryonic, larval, and juvenile stages, and during posterior regeneration.

## 2. Materials and Methods

### 2.1. Animals, Experimental Manipulations and Fixation

Epitoke individuals of *A. virens* were caught during spawning near the Marine Biological Station of SPbSU at the White Sea. Laboratory culture of embryos was obtained by artificial fertilization [26]. When embryos/larvae or juvenile worms reached the preferred stage, they were fixed in 4% PFA on 1.75× PBS with 0.1% Tween 20 at +4 °C overnight, then put in 100% MetOH for storage at −20 °C. In laboratory culture, the juveniles grew for 3 months in small aquariums with natural or artificial seawater at 18 °C. Procedures for caudal amputation and cultivation of regenerating juvenile worms have been described previously [30,32,33]. Before amputation of the posterior third of the juveniles’ body, the 18–20-segment long animals were anesthetized in a mixture of artificial seawater and 7.5% MgCl_2_ (1:1). Worms were washed in filtered artificial seawater and placed into clean, small Petri dishes. The samples were anesthetized again and fixed (see above) at the following time points: 12 h post-amputation (hpa), 1 day post-amputation (dpa), 2, 3, and 4 dpa.

### 2.2. Sequence Retrival and Phylogenetic Analysis

The sequence of Nanos was found in an unannotated transcriptome database for *Alitta virens* (local resource). Amino acid alignment of the nanos CCHC zinc finger domain was performed with MUSCLE 3.8.31 [34] using the Phylogeny.fr web server (accessed on 14 January 2022) [35]. Domain organization of the sequences was established using the online program PROSITE (https://prosite.expasy.org/ accessed on 1 December 2021). Bayesian phylogenetic analysis was conducted using the Markov Chain Monte Carlo method implemented in MrBayes 3.2.6 (http://www.phylogeny.fr/ accessed on 14 January 2022) [35,36,37]. The number of substitution types was fixed to 6. The Poisson model was used for substitution, while rates variation across sites was fixed to “invgamma”. Four Markov Chain Monte Carlo (MCMC) chains were run for 100,000 generations, sampling every 100 generations, with the first 250 sampled trees discarded as “burn-in”. Finally, a 50%-majority-rule consensus tree was constructed. The phylogenetic tree was handled using the FigTree program 1.4.4 (http://tree.bio.ed.ac.uk/software/, accessed on 14 January 2022).

### 2.3. Gene Cloning

In order to search for possible additional genes, degenerate PCR with larval *A. virens* cDNA was performed. The sequences for the selected degenerate primers are as follows: upstream primer 5′-TGYGTNTTYTGYMRNAMNAA-3′ and downstream primer 5′-GGRCARTAYTTDATNGTRTG-3′. No additional *nanos*-related gene fragments were identified. For RNA probes for in situ hybridization, a fragment of candidate sequence was isolated by gene-specific PCR and cloned. The sequences for the gene-specific primers are as follows: upstream primer 5′-GTTGTACGGAGATTGGAATCATTGG-3′ and downstream primer 5′-GCAACTAGGTCACACGACAGATG-3′. The amplified fragment was 1366 bp in length and included 774 bp of open reading frame (ORF), 48 bp 5′UTR, and 544 bp 3′UTR. The PCR product was inserted in a pCRII vector by using TOPO-TA cloning (Invitrogen) and used for the transformation of chemically competent *E. coli* (One Shot™ TOP10). When colonies with the correct insert were obtained and checked by sequencing, digoxigenin-labeled RNA probes (antisense and sense) were synthesized and used for in situ hybridization.

### 2.4. Whole-Mount in Situ Hybridization

The whole-mount in situ hybridization (WMISH) experiments were performed as described previously [33]. Objects were rehydrated from MetOH then rinsed in PTW, treated with proteinase K (100 µg/mL) for 0.5–2.5 min at +22 °C, twice rinsed in glycine (2 mg/mL), postfixed with 4% PFA on PTW for 20 min. The samples were washed in PTW before the pre-hybridization step. After incubation with the DIG-probe, subsequent washes, overnight incubation with anti-digoxigenin AP antibodies (dilution 1:2500), and washing, the objects were stained with NBT/BCIP, followed by washing in PTW and mounting in 90% glycerol. In situ hybridization with the sense, DIG-labeled riboprobe was used as a negative control (Supplementary Materials Figure S1).

### 2.5. Data Visualization

Results of in situ hybridization were visualized using DIC optics with an Axio Imager D1 microscope (ZEISS, Oberkochen, Germany). The figures were made in Adobe Illustrator.

## 3. Results

### 3.1. Sequence Analysis

**Figure 1:**
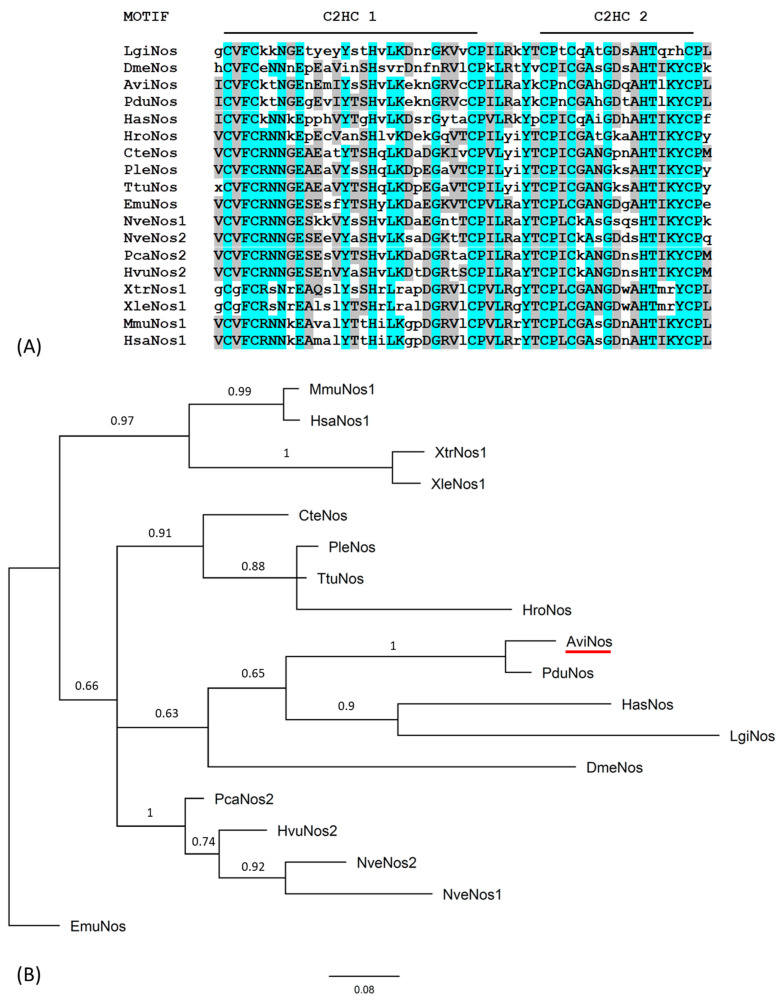
Sequence alignment and phylogenetic analysis of *nanos* gene from *Alitta virens*. (**A**) Multiple sequence alignment of AviNos (*underlined in red*) with other Nanos zinc finger domain sequences from vertebrates and invertebrates. (**B**) Phylogenetic analysis of *Alitta virens nanos* gene. Bayesian consensus tree of the CCHC zinc finger domain of metazoan *nanos* genes: AviNos (*Alitta virens*, GenBank OL456152); CteNos (*Capitella teleta*, DAA06318.1); DmeNos (*Drosophila melanogaster*, AAA28715.1); Emu (*Ephydatia muelleri*, AJE59349.1); HasNos (*Haliotis asinine*, ACT35656.1); HroNos (*Helodbella robusta*, AAB63111.1); HsaNos1 (*Homo sapiens*, NP_955631.1); HvuNos2 (*Hydra vulgaris*, BAB01492.1); LgiNos (*Lottia gigantean*, XP_009044182.1); MmuNos1 (*Mus musculus*, NP_848508.2); NveNos1 (*Nematostella vectensis*, AAW29070.1); NveNos2 (*Nematostella vectensis*, AAW29071.1); PcaNos (*Podocoryne carnea*, AAU11514.1); PduNos (*Platenereis. Dumerilii*, CAJ28985.1); PleNos *(Pristina leidyi*, ADE44350.1); TtuNos (*Tubifex tubifex*, BAQ21630.1); XleNos1 ((*Xenopus laevis*, NP_001081503.1); XtrNos1 (*Xenopus tropicalis*, NP_988857.1).

A single *nanos* fragment was identified in the *A. virens* transcriptome and isolated by gene-specific PCR from *A. virens* cDNA (mixed-stage regeneration); the cDNA sequence is available from GenBank under accession number OL456152. The full-length *Avi-nanos* transcript encodes a putative protein of 257 amino acids. The *Avi-nanos* gene possesses two CCHC zinc finger domains that display the highest degree of amino acid identity with other metazoan zinc finger domains from Nanos proteins (Figure 1A). Phylogenetic analysis, based on amino acid alignment of the two CCHC zinc finger domains of the metazoan Nanos proteins, clusters Avi-Nanos with the Nanos from *Platynereis* and other lophotrochozoan. Obviously, Avi-Nanos is more closely related to vertebrate, cnidarian, and Drosophila sequences than to the Nanos protein of the sponge *Ephydatia* (Figure 1B). No additional *nanos*-related genes were isolated by degenerate PCR from larval *A. virens* cDNA. Thus, it is most likely that *A. virens* has a single *nanos* gene.

### 3.2. Avi-nanos mRNA Larval and Juvenile Expression Patterns

**Figure 2:**
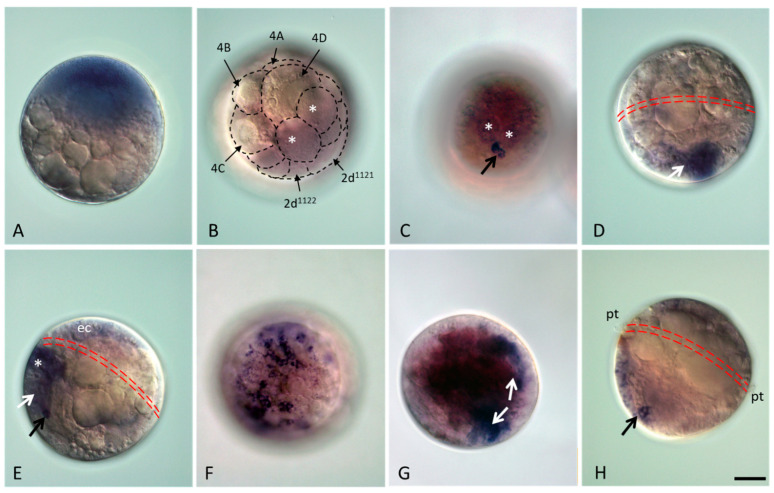
*Avi-nanos* RNA expression patterns during embryonic and trochophore larva development. (**A**) Zygote after completion of ooplasmic segregation. *Avi-nanos* RNA is detected in the yolk-free cytoplasm of the animal hemisphere. (**B**) Cleavage stage. *Avi-nanos* transcript is observed in almost all blastomeres, including the 4d daughter cell: *white asterisks* indicate mesoteloblast; *black dotted lines* highlight the blastomeres. (**C**) Early gastrulation stage, superficial vegetal view. Strong expression is observed in small cells, putative primordial germ cells (PGCs) (*arrow*), descendants of mesoteloblasts (*asterisks*). (**D**) Mid-gastrula stage, lateral view, ventral on the left, animal pole facing up. Expression in the mesoteloblasts and forming mesodermal bands (*arrow*). The position of the presumptive prototroch is marked by *double red dotted line*. (**E**) Late gastrulation–early trochophore, lateral view in a deep focal plane, ventral on the left, animal pole upward. Expression in the mesodermal bands (*white arrow*), stomodeal cells (*asterisk*), epidermal cells of the episphere (prospective brain cells, *ec*), and putative PGCs (*black arrow*). The position of the presumptive prototroch is marked by *double red dotted line*. (**F**) Trochophore, anterior view in a surface focal plane, ventral to the left. *Avi-nanos* expression in the presumptive brain. (**G**) Trochophore, anterior-ventral view in a deep focal plane. Expression in the bilateral mesodermal bands (*arrows*). (**H**) Late trochophore, lateral view in a deep focal plane, ventral on the left. Expression in the ventral plate (hyposphere), hindgut region, including putative PGCs (*arrow*), and epidermal cells of the episphere. The position of the prototroch is marked by *double red dotted line*: *pt*, ciliated cells of the prototroch. Scale bar, 40 mkm for all panels.

*Avi-nanos* expression was examined by WMISH throughout embryonic and larval development, metamorphosis, and at several growth stages of juvenile *A. virens*. *Avi-nanos* transcript is detected in the yolk-free cytoplasm in zygotes. After the completion of ooplasmic segregation, *Avi-nanos* mRNA is found in the animal hemisphere (Figure 2A) of the zygotes. Through early cleavage stages, the transcript is detected in most, if not all, blastomeres of all four embryonic quadrants (Figure 2B). Later, expression of *Avi-nanos* significantly decreases, and strong expression is only observed in few very small cells (4d daughter cells), although it is also detected at much lower levels throughout the embryo (Figure 2C). As mesodermal band formation progresses, the level of the transcripts becomes noticeably higher in the 4d descendant cells (Figure 2D,E). At the end of gastrulation, *Avi-nanos* expression is observed in a more discrete pattern with four distinct domains, including epidermal cells of the hyposphere, the presumptive brain, the closing blastopore, and mesodermal cells (Figure 2F–H).

**Figure 3:**
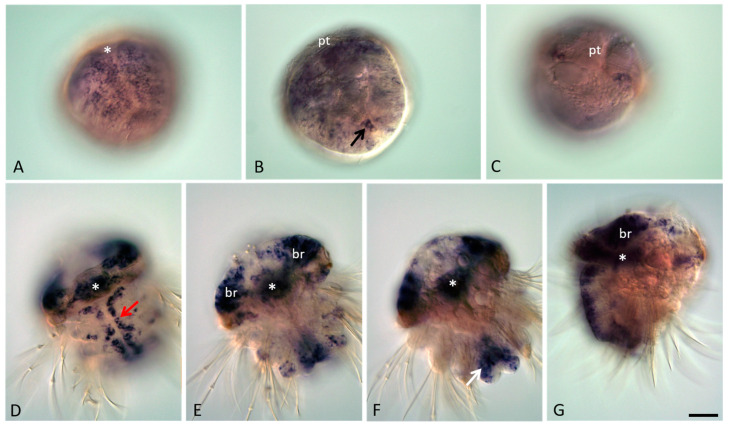
*Avi-nanos* RNA expression patterns during metatrochophore larva development. Except for (**C**,**G**), all animals are oriented anterior to the upper left corner. (**A**–**C**) Early metatrochophore at different focal planes; (**D**–**G**) Late metatrochophore at different focal planes. (**A**) *Avi-nanos* is broadly expressed in most cells of the ventral plate, including the forming stomodeum (foregut anlage) and in the presumptive neuroectoderm; ventro-caudal view in a surface focal plane. (**B**) A *nanos* transcript is observed in the developing chaetal sacs and proctodeum (hindgut anlage), where putative PGCs are located (*arrow*); ventro-caudal view, deeper. (**C**) Dorsal surface view, anterior upward. (**D**) Robust *Avi-nanos* expression is detected in the stomodeum, ventral nerve system, and putative peripheral neurons; ventral view in a surface focal plane. (**E**) Expression in the brain, stomodeum, and the putative parapodial neurons; ventral view, deeper. (**F**) Expression in the anal lobes and the hindgut (*arrow*). (**G**) A deep lateral view of the late metatrochophore, anterior upward, ventral to the left. *white asterisk* mark position of stomodaeum: *br*, brain, *pt*, prototroch. Scale bar, 40 mkm for all panels.

**Figure 4:**
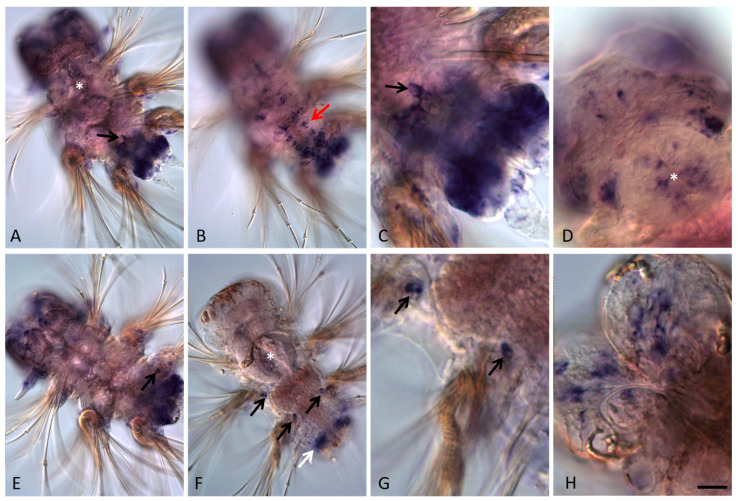
*Avi-nanos* RNA expression patterns during nectochaete larva development and metamorphosis into a four-segmented juvenile worm. All animals are oriented anterior to the upper left corner. (**A**–**D**) Nectochaete at different focal planes; (**E**–**H**) juvenile animals at different focal planes. (**A**) *Avi-nanos* is broadly expressed in most caudal area that corresponds with the future posterior growth zone, and in the putative PGCs (*arrow*). (**B**) Expression in the ventral nerve cord (*arrow*); ventral view in a more surface plane. (**C**) Enlarged view of the caudal region of the nectochaete shown in (**A**); dorsal surface view, anterior upward. (**D**) Expression in the pharynx and in the head; view from dorsal side. (**E**,**F**) Expression in the growth zone and the next-forming segment (*white arrow*). Migrating PGCs (*black arrows*); deep ventral view. (**G**) Enlarged view of the migrating PGCs shown in (**F**). (**H**) Expression in the brain. * marks the pharynx. Scale bar, 40 mkm (**A**,**B**,**E**,**F**); 13 mkm (**C**,**G**,**D**); or 16 mkm (**H**).

From the trochophore stage when the ventral plate is formed in the hyposphere, a robust expression of *Avi-nanos* is observed in the anlage of the ventral nerve cord. During metamorphosis of the trochophore into the metatrochophore, the number of such cells increases significantly (Figure 3A). The midline expression domain is confined to the apical cell layers (Figure 3D,G). This expression is gradually restricted first to two bilateral cell domains at the ventral midline, which correspond to the subsets of the neuroblast cells (metatrochophore stages), and later to single cells in every segmental ganglion in nectochaete larvae (Figure 3D; Figure 4B).

*Avi-nanos* is expressed in the presumptive brain through larval development, including during the late nectochaete stage when the brain is morphologically distinct from the overlying ectoderm (Figure 3E,G; Figure 4A). Expression in the brain is reduced towards the end of metamorphosis of the nectochaete larva into a four-segmented juvenile worm, but it is still detected at later developmental stages (Figure 4D,E,H). *Avi-nanos* transcript is observed in the foregut during very early stages of foregut development; it is found in cells that correspond to some cells around the closing blastopore at the early trochophore stage (not shown). Robust foregut expression is detected at the metatrochophore stages (early, middle, and late metatrochophore) and at low levels at the nectochaete stage (Figure 3D–G; Figure 4D). *Avi-nanos* is also expressed for a short period in the hindgut during the early and middle metatrochophore stages (Figure 3F,G). At these stages, the pygidial lobes start to form, almost all cells of which show *nanos* transcripts. A set of *Avi-nanos*-positive cells are also apparent immediately anterior to the ciliated telotroch at the posterior end of the larvae. These cells are ectodermal cells that form a band around most of the circumference of the larva. The position of these cells corresponds to the future posterior growth zone.

**Figure 5:**
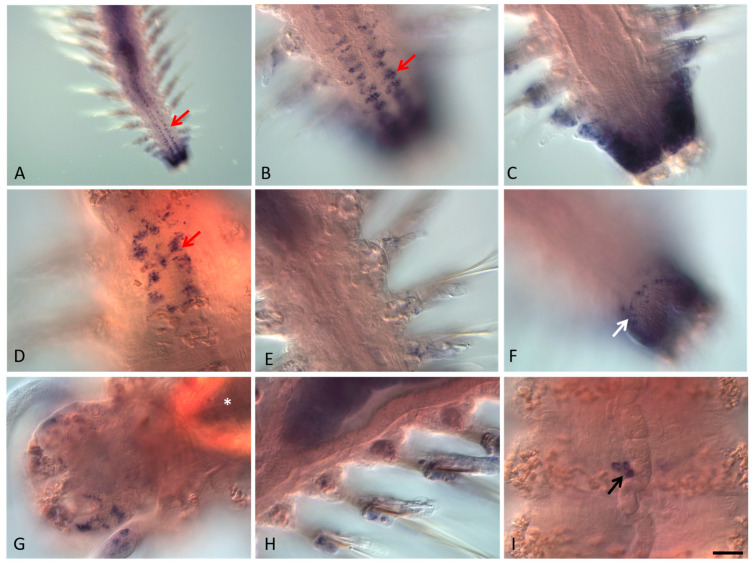
*Avi-nanos* RNA expression patterns in the 18-20 segment-long juvenile worms. Except for (**G**,**H**), all animals are oriented anterior upward; in (**G**,**H**) anterior is down and left. (**A**) *Avi-nanos* is broadly expressed in the posterior growth zone, newly developed segments, and the ventral nerve cord (*arrow*); ventral view. (**B**) Enlarged view of the caudal region of the animal shown in (**A**); (**C**) image (**B**) in a different focal plane. (**D**) Expression in the cells of the ventral nerve cord (*arrow*), anterior segments; ventral view. (**E**) Expression in the parapodia, ventral view. (**F**) Expression in epidermal cells of the growth zone (*arrow*); dorsal view. (**G**) Expression in the head and the peristomial cirri; dorsal view. (**H**) Expression in the parapodia; dorsal view. (**I**) A cluster of *nanos*-expressing cells (PGCs) located right behind the jaws (*arrow*). * marks the pharynx. Scale bar, 40 mkm for all panels except (**A**). Scale bar in (**A**), 85 mkm.

At the end of larval development, four small, roundish *nanos*-positive cells emerge from the posterior (hindgut) region and begin migrating anteriorly (Figure 4A,C,F,G). During the 4–5-segmented juvenile worm stage these cells can be found in various positions, usually close to the parapodia base, but they are never observed in the cephalic segment, which will later be integrated into the head. A cluster of four *nanos*-expressing cells located right behind the jaws (segments 4–5) is detected in 18–20-segment long juvenile worms (Figure 5I). All juveniles studied in this work (4–5 and 18–20-segment long animals) are characterized by robust *Avi-nanos* expression in the posterior growth zone and in the newly formed postlarval segments (Figure 5A–C,F). The number of the *nanos*-positive cells (both superficial and internal) is especially high on the ventral side. Expression of *Avi-nanos* is also detected in the ventral cells of the nervous system in almost all body segments in juvenile *A. virens* (Figure 5A,B,D) and in the individual cells of both the ventral and dorsal parts of parapodia (Figure 5E,H). Domains of *Avi-nanos* mRNA localization are found in the head, including eye regions, and the peristomial cirri (Figure 5G).

### 3.3. Avi-nanos Expression during Regeneration after Caudal Amputation

**Figure 6:**
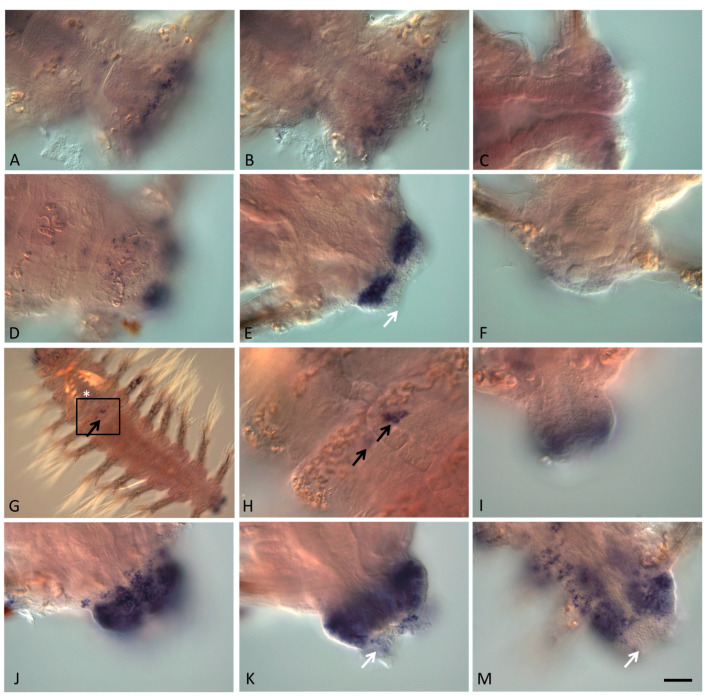
*Avi-nanos* RNA expression patterns during posterior regeneration. Except for (**C**,**F**), all animals are oriented anterior to the upper left corner; in **C** anterior is to the left; in **H** anterior is up. The pictures show the following stages of regeneration: (**A**–**C**) 12 hpa; (**D**–**F**) 2 dpa; (**G**–**K**) 3 dpa; (**M**) 4 dpa. (**A**) *Avi-nanos* is expressed in cells at the wound site; ventral view. (**B**) The same animal; deeper focal plane. (**C**) Dorsal view, deep. (**D**) Expression in the neural ganglia cells; ventral view. (**E**) A robust expression of *nanos* is detected in the epidermal and blastemal cells of the regeneration bud, except for the most terminal dorsal region of the pygidium anlage (*arrow*); ventral view, deeper. (**F**) Expression in epidermal cells of the forming growth zone; dorsal view. (**G**) Expression in the head, parapodia, regeneration bud, and in a cluster of four *nanos*-expressing cells (PGCs; *arrow*) located right behind the jaws (denoted by *). (**H**) Enlarged view of the *boxed region* shown in (**G**) highlighting a cluster of PGCs (*arrows*). (**I**) Expression in epidermal cells of the forming growth zone; dorsal view. (**J**,**K**) Expression during the beginning of the first restored segment formation; ventral view, different focal planes. A weak expression persists in growing pygidial cirri (*arrows*). (**M**) Progression of regeneration is accompanied by increased *Avi-nanos* expression in the cells of the nerve ganglia of the surviving segments adjacent to the amputation plane; ventral view. Expression disappears in the new pygidium (*arrows*). Scale bar, 40 mkm for all panels except (**G**). Scale bar in (**G**), 85 mkm.

In experimental conditions, after amputation, *A. virens* juveniles are able to restore the pygidium, growth zone and the lost segments after several days. Although the complete regeneration takes 5 to 10 days, formation of the first restored segment usually occurs 4 dpa. At the site of the amputation, *Avi-nanos* expression is detected 12 hpa, before the completion of wound healing. It appears *de novo* in both superficial cells and internal cells, mostly on the ventral site of the wound (Figure 6A, B). Obviously, no *nanos* transcripts are visible in the injured gut epithelium (Figure 6C). Later, at the 1 dpa stage, when the wound healing is complete, expression of *Avi-nanos* is found in the forming posterior blastema (not shown). At the 2 dpa stage, domains of both the ventral epidermal and internal *nanos*-positive cells grow significantly (Figure 6D,E). At this point in time, the epidermal cells on the dorsal side of the regeneration also show expression, but at a very low level (Figure 6F).

During the next day of the experiment, the regeneration bud shows noticeable growth due to active proliferation of the epidermal and blastemal cells, especially on the ventral and lateral sides [31,32]. At this stage, *Avi-nanos* mRNA is detected in almost all cells of the regeneration bud, except for the most terminal end, where the anal cirri form (Figure 6J,K). Although the level of *Avi-nanos* in most dorsal cells is still very low, there are some cells that are characterized by increased transcript levels (Figure 6I). These dorsal cells, together with the ventral and lateral cells, form a new posterior growth zone. Already at the 3 dpa stage, especially dense domains of *nanos*-positive cells adjacent to the surviving posterior segment (Figure 6K) become visible, and a new segment begins to develop. 

Interestingly, during regeneration, *Avi-nanos* mRNA can be observed in the ventral nerve cord ganglia of almost all segments, however, the number of *nanos*-positive cells decreases markedly (Figure 6D). Nevertheless, in the two days after initiation of the development of the first new segment, expression in the ventral nervous system again increases significantly (Figure 6M). The cluster of *nanos*-expressing cells located right behind the jaws retains its position and composition throughout the period of pygidium recovery, growth zone formation, and the beginning of production of new segments (Figure 6G,H). Meanwhile, the character of *Avi-nanos* expression in the parapodia and in the head remains unchanged (Figure 6G). No signs of migration of *nanos*-expressing cells were found.

## 4. Discussion

In this work an ortholog of the *nanos* gene family (*Avi-nanos*) was isolated from the annelid *A. virens*, and its developmental expression patterns were examined by whole-mount in situ hybridization during the embryonic, larval, and juvenile stages, and during posterior regeneration. It has been shown that a *nanos* homolog is expressed in both germline and in multiple somatic tissues during *A. virens* development. *Avi-nanos* mRNA is shown in the brain, ventral nerve cord, foregut, hindgut, mesodermal bands, and posterior growth zone, and appears de novo in blastema during regeneration.

Similar to most lophotrochozoans that have been studied (except for *P. dumerilii*, s. below), *Avi-nanos* transcript is detected in the yolk-free cytoplasm in uncleaved zygotes, which indicates maternal expression without specific asymmetric localization [18,19,20,23,38,39,40]. Further distribution of the *nanos* gene product in most blastomeres of all four quadrants suggests the absence of the effect of ooplasmic segregation [27,28] on the inheritance of the maternal transcript by specific blastomeres. Similar to *Capitella* [23], expression of *Avi-nanos* is enriched in 4d daughter cells at the fifth to sixth cleavage, although it is also detected at lower levels throughout the embryo. This pattern contrasts with the restricted *nanos* expression at a similar stage in the snail, *T. obsoleta* [18]. Indeed, during the first five cleavage cycles, *nanos* mRNA is found in all cells of the *Tritia* embryos. However, between the 24- and 36-cell stages, *IoNanos* mRNA becomes restricted to two cells: the yolk-rich 4d macromere and the 4d micromere; later it is specifically localized in the mesendodermal blast cells derived from the 4d blastomere. On the other hand, a broad expression of a *nanos* homolog is found in the early embryo of the vetigastropod *H. asinine*. *HasNanos* expression is detectable during embryonic and larval development—being maternally expressed it is localized to the micromere cell lineages during early cleavage [19]. In the annelid *A. virens*, at the sixth to seventh cleavage, a robust expression is detected in very small cells, although it is also detected at lower levels throughout the embryo. These cells are products of the first cell divisions of the mesoteloblasts (4d daughter blastomeres) [41] and are putative PGCs [41,42,43,44,45]. A broad *Avi-nanos* expression encompasses all 4d descendants, not only those which will give rise to the primordial germ cells but also the major derivative of mesoteloblasts: segmental mesoderm, which includes body wall, visceral muscle, and coelomic cavity linings. In the oligochaete annelid *Tubifex tubifex*, a broad expression of *Ttu-nos* throughout the early cleavage stages (up to the 22-cell stage) becomes restricted to 2d and 4d cells. After teloblast formation, it is detected in nascent primary blast cells produced by mesodermal teloblasts M and ectodermal teloblasts N and Q [40]. In the glossiphoniid leech embryo (*Helobdella robusta*), a high level of Nanos protein was detected in ectodermal precursor cells during cleavage, but with lower expression levels in mesodermal precursors. Thus, the leech *nanos* lacks restricted expression in the 4d lineage [38]. 

When the trochophore develops, *Avi-nanos* mRNA is shown to be expressed in the putative mesodermal bands of the larva, similar to *Capitella*, *Platynereis*, *Tritia,* and *Haliotis*, and in the PGCs, similar to *Capitella*, *Platynereis* and *Helobdella* [18,19,20,22,23]. Interestingly, further *nanos* expression in polychaetes is not restricted to mesodermal derivatives. Strong expression is shown in the epidermal and neural structures, the forming foregut and hindgut, parapodia cells, and the growth zone, including its ectodermal component [22,23] and this study. During metamorphosis of the *A. virens* trochophore into the metatrochophore, when the larval body extends along the anterior–posterior axis by ventral plate growth due to convergence of cells from lateral sides, as well as by proliferation within the ventral plate itself, the epidermal domain of *Avi-nanos* expression is especially broad. Later, at the nectochaete stage, it becomes more restricted to the forming growth zone. *Avi-nanos*-positive putative PGCs migrate anteriorly through the parapodia base at the late nectochaete–juvenile stage. Positions of the *Avi-nanos*-expressing PGCs in nectochaete and juvenile stages vary to a high degree. Later, they form a cluster of four *nanos*-expressing cells located right behind the jaws (segments 4–5). In the first study on *Platynereis*, the *Pdu-nanos* transcript was not detected in the zygote, during early cleavage, or at the juvenile stages [22]; most likely this was due to technical issues. Later, a strong *Pdu*-*nanos* gene expression was shown in juveniles and regenerates of *P. dumerilii* [24,25]. Obviously, the maternal phase of gene expression, as well as a varied pattern of zygotic expression in both the germline and somatic cells, is a common feature of annelid development. 

Expression of *nanos* homologs during annelid regeneration has not been adequately studied. In the oligochaete *Pristina leidyi*, *PRIle-nanos* is strongly expressed in the posterior growth zone. Its expression appears de novo in the fission zone during asexual reproduction and in the blastema cells during anterior and posterior regeneration [46,47]. In *Alitta*, as well as in *Platyneres*, the *nanos* transcript appears de novo at the wound site during the early stages of regeneration [this study, 25]. According to our previously published data, other GMP genes (*Avi-pl10*, *Avi-vasa*, *Avi-piwi1*, and *Avi-piwi2*) also start their expression de novo at the injury site during *A. virens* posterior regeneration [32]. Transcripts of *pl10*, *vasa*, *piwi*, and *nanos* homologs are detected at all steps of caudal regeneration. Similar to *Avi-pl10*, *Avi-vasa, and Avi-piwi1*, *Avi-nanos* mRNA is broadly distributed across the whole regeneration bud, while *Avi-piwi2* signal is found exclusively in the blastema. In *Alitta*, expression of the studied GMP genes becomes especially strong during blastema growth and differentiation (2–3 dpa). This period is characterized by active cell proliferation across the regeneration bud and formation of the new growth zone [30,31,32]. Later, when new segment primordia have become apparent, expression of *Avi-vasa* and *Avi-piwi2* gradually fades in the nascent segments, while *Avi-nanos*, *Avi-pl10*, and *Avi-piwi1* are still broadly expressed in the regenerating posterior end of the worm. These differences in gene expression correspond to observed patterns in unamputated, growing juvenile worms [32] this study. In contrast to other GMP genes from *A.virens*, *Avi-nanos* is also detected in the ventral nerve cord ganglia of almost all segments, although the number of *nanos*-positive cells decreases markedly during the first few days of regeneration. No signs of *nanos*-positive cell migration were found, and the cluster of *nanos*-expressing PGCs located right behind the jaws remains unchanged. The obtained results show the expression of a pluri-/multipotent stem cell marker (*nanos*) in the blastema from an early stage. These data also support the opinion that blastemal cells are mostly derived from cells of the segment adjacent to the amputation site, and that the cluster of PGCs is not required for regeneration in the nereid polychaetes [24,32,45].

Local cell dedifferentiation, rather than the migration of stem cells, is considered the source of the blastema in most polychaetes [32,48,49]. However, the molecular signals that induce the blastema precursor cells in annelids have not been identified yet. Our recently published data suggest that FGF is essential for blastema formation and regeneration in *A. virens* [31]. At the 4 hpa stage, expression of the FGF genes is already found in the surface cells, nervous system and mesodermal cells at the injury site. In *A. virens*, FGF inhibitors prevent regeneration, most likely by suppression of cell proliferation and blastema initiation. On the other hand, GMP genes (*vasa*, *pl10*, *piwi*, and *nanos*) have an extensive expression in FGF-competent tissues starting at day 2 of *A. virens* posterior regeneration. This suggests that FGFs in *A. virens* may also affect premature cell differentiation during blastema growth and patterning [31]. Thus, FGF signals are possible candidates for reprogramming cell fates in annelid regeneration. Further studies will show if FGF inhibitors affect expression of *nanos* and other GMP genes during *A. virens* regeneration.

## Data Availability

mRNA sequence of *Avi-nanos* is deposited in GenBank with the accession number OL456152.

## Supplementary Materials

The following supporting information can be downloaded at: https://www.mdpi.com/article/10.3390/genes13020270/s1, Figure S1: Results of in situ hybridization with the sense *Avi-nanos* DIG-labeled probe (negative control).
