# Supplementary material for: Nanos Is Expressed in Somatic and Germline Tissue during Larval and Post-Larval Development of the Annelid Alitta virens
Source: Genes (Basel). 2022 Jan 29;13(2):270. doi: 10.3390/genes13020270 (PMC8871563; doi:10.3390/genes13020270)
Supplement: Supplementary file 1 [file genes-13-00270-s001.zip › genes-1560156-supplementary.pdf]

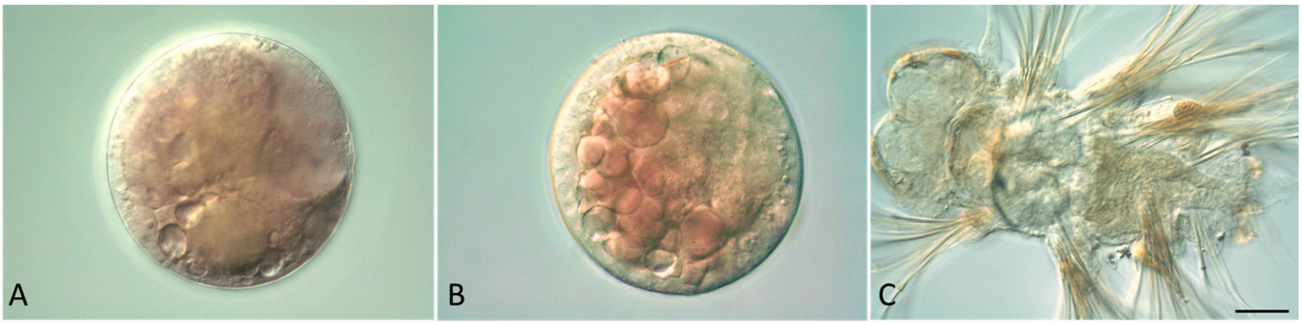

Figure S1. Results of in situ hybridization with the sense *Avi-nanos* DIG-labeled probe (negative control). **A**–Cleavage stage. **B**–Trochophore stage. **C**–4-segmented juvenile worm. Scale bar, 40 mkm for all panels.
